# Assaying and classifying T cell function by cell morphology

**DOI:** 10.3390/biomedinformatics4020063

**Published:** 2024-04-26

**Authors:** Xin Wang, Stacey M. Fernandes, Jennifer R. Brown, Lance C. Kam

**Affiliations:** 1 Department of Biomedical Engineering, Columbia University, New York, NY; 2 Department of Medical Oncology, Dana-Farber Cancer Institute, Harvard Medical School, Boston, MA

**Keywords:** T cell morphology, intrinsic state, surrounding environment, primary human T cells, CLL, T cell mechanosensing, rapid measurement, immunotherapy, machine learning

## Abstract

Immune cell function varies tremendously between individuals, posing a major challenge to emerging cellular immunotherapies. This report pursues the use of cell morphology as an indicator of high-level T cell function. Short-term spreading of T cells on planar, elastic surfaces was quantified by 11 morphological parameters and analyzed to identify effects of both intrinsic and extrinsic factors. Our findings identified morphological features that varied between T cells isolated from healthy donors and those from patients being treated for Chronic Lymphocytic Leukemia (CLL). This approach also identified differences between cell responses to substrates of different elastic modulus. Combining multiple features through a machine learning approach such as Decision Tree or Random Forest provided an effective means for identifying whether T cells came from healthy or CLL donors. Further development of this approach could lead to a rapid assay of T cell function to guide cellular immunotherapy.

## 1. Introduction

Cells of the immune system execute and coordinate a wide range of functions in both normal and pathological physiologies. These cells are also a compelling platform for targeted, effective, and persistent therapy for a range of diseases, seen most prominently in the successful clinical deployment of T cells against cancer [1–4]. However, the use of cells as a “living drug” poses several challenges, including wide variability in functionality between individuals as a result of disease state. In the case of T cell therapy against cancer, long-term diseases such as Chronic Lymphocytic Leukemia (CLL) induce T cell deficiencies resembling cellular exhaustion, complicating the preparation of therapeutic quantities of cells, and ensuring efficacy once reintroduced to the patient [5–11]. The ability to rapidly estimate the responsiveness of an individual’s T cells would dramatically improve cell production by tailoring *ex vivo* culture conditions to an individual’s starting material and provide powerful insight into T cell health over the course of treatment. Current assays in this direction include cell count, biomarkers, and cytokine secretion. However, our group demonstrated that assays of high-level cellular function, specifically cell migration, provide enhanced insight into long-term cell function compared to these molecular measures alone [12].

This report examines cell morphology as a simpler indicator of T cell functionality, reflecting both the intrinsic state of an individual’s cells as well as response to the surrounding environment. In these assays, T cells are allowed to interact with planar test surfaces under controlled *ex vivo* conditions. Like other cells, T cells undergo a phase of rapid spreading, driven by actin polymerization, followed by contraction of this cytoskeletal network [13,14]. The shape of cells on these surfaces reflects a balance of intracellular processes and the interaction of cells with the extracellular environment. Consequently, measures of cell spreading, such as area, have been used as surrogates of T cell activation and subsequent function [15,16]. In this study, we refine this basic approach to capture changes in cell morphology as a function of two different types of factors. As a cell-intrinsic factor, we compare T cells isolated from healthy donors against counterparts from patients being treated for chronic lymphocytic leukemia (CLL), a disease often accompanied by T cell exhaustion. As a complementary, extrinsic factor, we examine cell response to substrates of different mechanical stiffness. This is inspired by a growing body of knowledge that T cells respond to the mechanical resistance of their environment, altering a range of readouts from cytokine secretion to long-term proliferation [17–19]. This study examines how morphological outputs can be associated with these two factors, providing a measure of the overall cellular states.

## 2. Materials and Methods

### Glass-supported Poly(dimethyl siloxane)(PDMS) substrate preparation.

PDMS substrates of varying stiffness were prepared following established protocols [20,21]. Blending Sylgard 527 and Sylgard 184 in mass ratios of 10:1, 3:1, and 1:3 produced substrates with Young’s modulus of 250 kPa, 1000 kPa, and 2000 kPa. Thin PDMS layers were created on glass coverslips (thickness #0 Fisherband). A droplet of PDMS mixture was pressed using a PDMS cube to flatten the droplet and create a thin (~ 20 μm) layer, which would be peeled off after curing overnight at 65 °C. The PDMS cubes were silanized overnight with (tridecafluoro-1,1,2,2,-tetrahydrooctyl)-1-trichlorosilane (United Chemical Technologies), to facilitate removal from the PDMS substrates. To activate T cells, each PDMS substrate was coated overnight at 4 °C with a mixture of α-CD3 (clone OKT3, Bio X Cell) and α-CD28 (clone 9.3, Bio X Cell) antibodies in a mass ratio of 1:1 for a total concentration of 20 μg/mL in PBS. For visualization and quantification of surface-bound protein, half the antibodies in this mix was labeled with a fluorescent dye (Alexa Fluor 568).

### PDMS substrate stiffness characterization.

Young’s modulus (E) of prepared PDMS substrates was measured by indentation [22]. Thick slabs (several mm) of PDMS were deformed with a flat cylindrical head and using a calibrated mass. The material’s Young’s modulus was estimated from the head diameter (D, 12 mm), deflection (h), weight (m), gravitational constant (g), and Poisson ratio (v) of 0.5 assuming Hertzian contact.


$$E=1-v2*m*\left(gD*h\right)$$

### Cell isolation and culture.

Mixed CD4^+^/CD8^+^ primary human T cells were isolated from Leukapheresis packs derived from healthy adult donors (New York Blood Center) and CLL patients (Dana-Farber Cancer Institute), using negative selection (RosetteSep kit, Stem Cell Technology) and gradient centrifugation (Ficoll-Paque PLUS, GE) [12]; cells were not purified on the basis of subtype, and consequently these preparations contained a mix of naïve, memory, and effector phenotypes. Cells were cultured in complete culture media consisting of RPMI 1640 (Thermo) supplemented with 10 mM HEPES (Gibco), 10 mM L-Glutamine (Gibco), 10% (v/v) fetal bovine serum (FBS; Gibco), 0.34% (v/v) β-mercaptoethanol (Sigma-Aldrich), and 10 mM penicillin-streptomycin (Gibco). After isolation, cells were frozen in complete media with 40% FBS and 10% DMSO in liquid nitrogen. Before experiments, cells were thawed and rested under standard culture conditions (37 °C, 5% CO2/95% air) overnight.

### Assays of cell spreading.

T cells were seeded onto glass-supported PDMS substrates at a concentration of 1×10^6^ cells/mL. Following 40 min T cell spreading, samples were fixed in 4% PFA for 20 min at room temperature and permeabilized with 0.1% Triton X for 10 min at room temperature. Then, samples were stained with Alexa Fluor 488 phalloidin (ThermoFisher) at 1:40 dilution for 20 min at room temperature, followed by washing twice. Samples were then imaged using an Olympus IX81 inverted microscope, equipped with an Andor iXon EMCCD camera, providing a 1002 × 1002 array of 8 μm × 8 μm pixels. Live imaging was conducted by live-cell microscopy under 60X magnification and bright field in the first 60 min after seeding T cells onto the PDMS substrate, using a stage top incubator (Tokai) [12]. The image was collected at 30-s intervals over the 60 min observation period. For analysis in live imaging, within the 120 frames, 2–5 cells were tracked and measured every 10 frames (5 min) by manual segmentation and measuring cell area on ImageJ. Fixed imaging was performed under 40X magnification to acquire more cells in a field of view for analysis. Image analysis was performed in ImageJ using functions of Smoothing, Thresholding, Set Measurement, and Analyze Particles to measure morphological features of single cells, including Area, Perimeter, Width, Height, Major, Minor, Circularity, Feret’s Diameter, Aspect Ratio (AR), Roundness, and Solidity [23]. Only accurately segmented single cells were selected by quality control for further analysis (Figure S2).

### Flow cytometry.

Flow cytometry to check the purity of isolated T cells was performed on a FACSCanto II (BD Biosciences) with a minimum of 10,000 gated events. Analysis was performed on FCS Express V6 (De Novo).

### Inhibitor studies.

Arp2/3 complex inhibitor CK666 (Sigma-Aldrich) (100 μM) was used to inhibit actin polymerization [24]. ROCK inhibitor Y-27632 (60 μM) was used to inhibit actomyosin contractility. Cells were pretreated with either CK666 or Y-27632 in complete culture media at 37 °C for 15 min and then were seeded onto the prepared PDMS substrates. The cells spread in the presence of the inhibitor for 40 min, followed by fixation, permeabilization, and incubation with Alexa Fluor 488 phalloidin, as previously described.

### Statistical analysis.

Unpaired t-tests with Welch’s correction and two-way ANOVA with Tukey multiple comparison test were conducted on GraphPad Prism 9.4.0 for quantitative comparisons of T cell Area and Roundness between Healthy and CLL, and across different PDMS stiffness conditions. Each data point in the bar graphs represents the average for that metric of all cells (approximately 100 cells) on a single PDMS sample, while data for all individual cells were included in analysis. Three healthy donors (H1, H2 and H3) and six CLL patients (D2, D18, D51, D63, D 67, D75) were included. For H1, n=4. For H2, n=8. For H3, n=6. For CLL patients, n=2 due to the limited availability of T cells in samples.

### Data preprocessing and data normalization.

Single-cell morphological features were acquired from spreading assays. These features include Area, Perimeter, Width, Height, Major, Minor, Circularity, Feret’s Diameter, Aspect Ratio (AR), Roundness, and Solidity. Only accurately segmented single cells were selected by manually labeling the segmentation quality with “good” or “bad.” There are 12,101 accurately-segmented single cells in total. The dataset was then aggregated into sample level – 100 samples in total. The values of morphological features were normalized using algorithm preprocessing.StandardScaler from scikit-learn (sklearn) library [25].

### Principal Component Analysis (PCA).

Principal Component Analysis (PCA) was performed on the normalized morphological feature dataset using the ‘PCA’ class from the ‘sklearn.decomposition’ module [25]. The first two principal components, which explain the majority of the variance, were retained to plot data [26,27].

### Feature-based classification.

Binary labels were assigned to Healthy (0) and CLL (1) samples. There are 45 CLL (1) samples and 55 Healthy (0) samples. The study employed three distinct classification models, namely Single-Feature Decision Tree, Multi-Feature Decision Tree, and Random Forest [28–30], to classify samples into Healthy or CLL. Decision Tree was performed using the algorithm DecisionTreeClassifier from the sklearn library [25]. For Single-Feature Decision Tree, only the feature Area was input for classification because Area was the primary feature we focused on in statistical analysis to compare Healthy and CLL. Similarly, Random Forest was performed using the algorithm RandomForestClassifier from the sklearn library [25]. Default hyperparameters were used as provided by the sklearn library to ensure reproducibility of our results across different studies. The models were compared through the average performance of three independent runs of 10-fold cross-validation. To prevent data leakage, each fold involved the random selection of one healthy donor and two CLL patients as the testing dataset. The evaluation metrics, including Accuracy, Area Under Curve (AUC), Sensitivity, Specificity, and Mathew’s correlation coefficient (MCC) were calculated to assess and compare the performance of the models.

### Study approval.

Primary human T cells were provided by the New York Blood Center or Dana-Farber Cancer Institute (under protocol 99-242). All cells are provided deidentified, without donor or patient identifying information; this study is exempt from DHHS regulations based on §46.104(d)(4).

## 3. Results

### 3.1. Disease state affects T cell morphology

**Figure 1.**
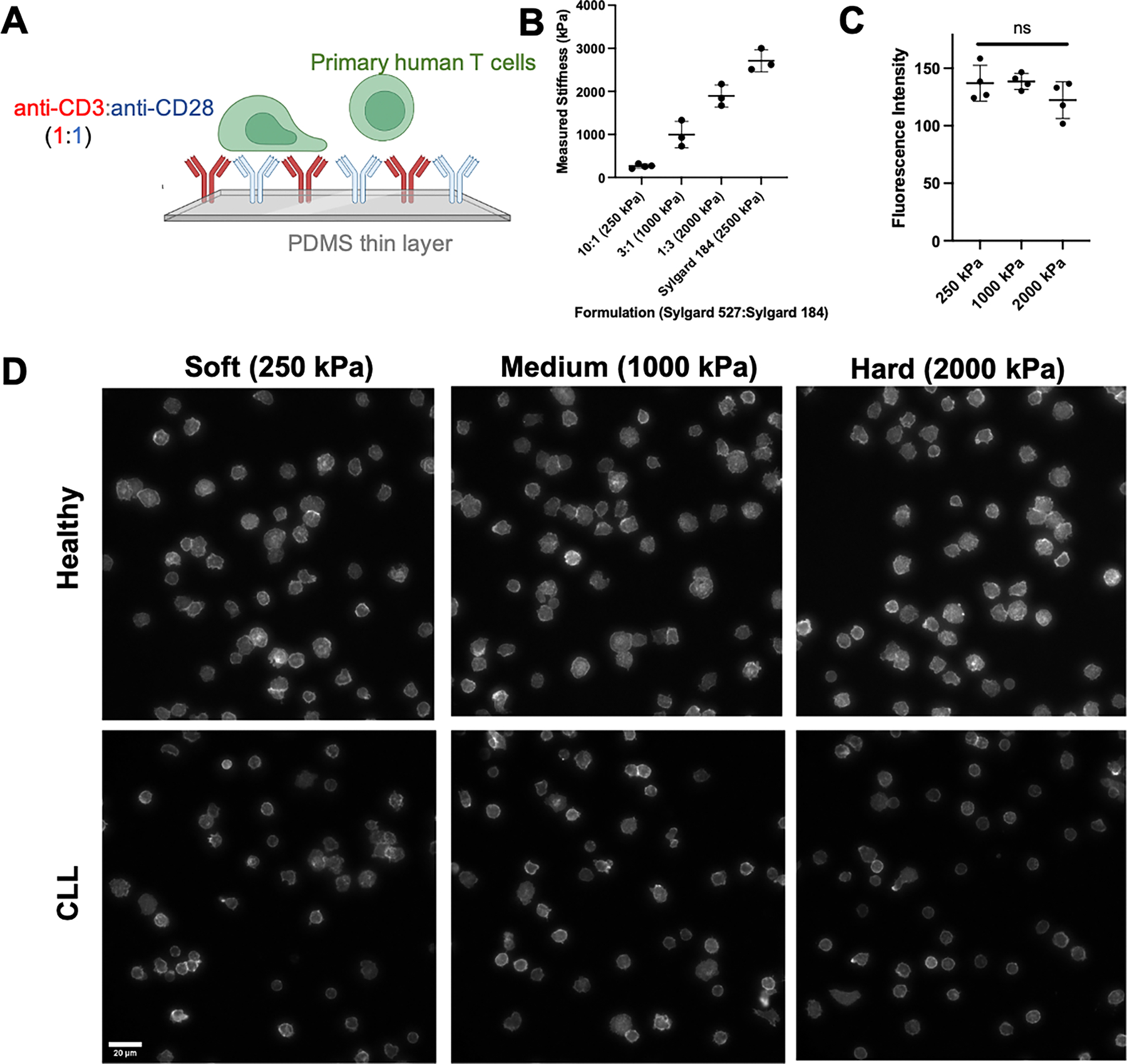
Characterization of PDMS substrates and visualization of T cell spreading from both healthy donors and CLL patients. (A) Schematic of antibody-coated PDMS thin layer to activate T cells. (B) Indentation testing was performed to measure the Young’s modulus of different PDMS formulations, with varying mass ratios of Sylgard 527 and Sylgard 184. Data are mean ± s.d., n=4 for 10:1(250 kPa), n=3 for the other formulations. (C) Quantification of antibody coating indicates a consistent level of OKT3 and 9.3 coated on the surfaces across different formulations of PDMS. Data are mean ± s.d., n=4 samples for each stiffness condition. (D) Fixed imaging finds that CLL T cells exhibit a smaller spreading area and a higher roundness than Healthy T cells, supporting the concept that disease state affects T cell morphology. Scale bar: 20 μm.

Primary human T cells from 3 healthy (H) donors and 6 CLL patients were allowed to spread on polydimethylsiloxane (PDMS) surfaces coated with α-CD3/CD28 (Figure 1A). These substrates were prepared by mixing two standard formulations of PDMS, Sylgard 527 and Sylgard 184. Elastic (Young’s) modulus was modulated by changing the ratio of the two formulations, producing three different stiffnesses of 250 (Soft, 10:1 ratio of 527:184), 1,000 (Medium, 3:1), and 2,000 (Hard, 1:3) kPa PDMS (Figure 1B). Comparison of the fluorescence intensity of Alexa 586-labeled OKT3/9.3 showed that the concentration of adsorbed antibodies was similar across the three different formulations (Figure 1C). Initial live-cell experiments (Figure S1A) captured the dynamics of cell spreading on these surfaces, identifying 40 min as a timepoint during which cell area, a representative measure of this interaction, stabilized on each surface and also produced the greatest difference as a function of elastic modulus. Toward a readily deployable assay of cell function, we focused on samples that were fixed at specific time points; the 40 min timepoint (Figure S1B) retained the stability and resolution seen in the live-cell assays and was chosen as a standard timepoint for the remainder of this study.

**Figure 2.**
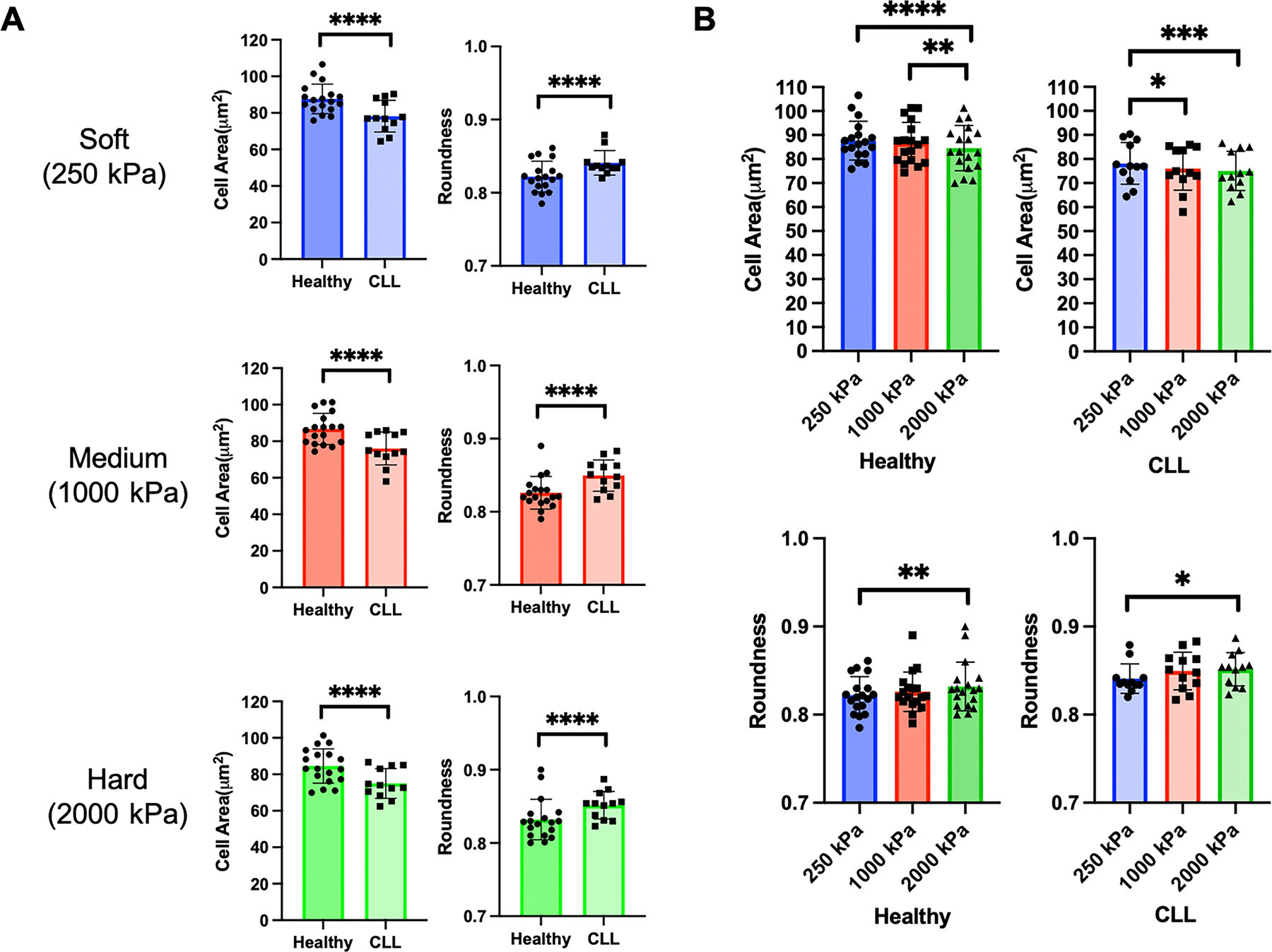
Quantitative analysis of T cell Area and Roundness from Healthy donors and CLL patients across three stiffness conditions. (A) CLL T cells show significantly smaller Area and higher Roundness than Healthy donors, and this applies to all three stiffness conditions. Data are mean ± s.d., each data point represents an individual substrate consisting of approximately 100 cells. Statistical significance was determined using unpaired t test with Welch’s correction across all cells captured for each condition, ****p<0.001. (B) T cells from healthy donors and CLL patients respond to substrate stiffness. Data are mean ± s.d., each data point represents an individual substrate consisting of approximately 100 cells. Statistical significance was determined using two-way ANOVA followed by Tukey multiple comparison test across all cells captured for each condition, *p<0.05, **0<0.01, ***p<0.005, ****p<0.001.

Representative images of fixed T cells from healthy donors and CLL patients on surfaces of different elastic modulus are shown in Figure 1D. Having been purified using techniques that are independent of subtype, these samples contained a mix of naïve, memory, and effector cells that are representative of the donor population. Most prominently, cells from CLL patients appear smaller than those from the healthy counterparts. This was confirmed by morphological analysis comparing cell Area across substrate stiffness (Figure 2A). Cell Roundness was also compared, recognizing that cells appeared to have different shapes across surfaces (Figure 1D). Notably, cells from CLL patients showed higher Roundness than cells from healthy donors (Figure 2A), supporting the concept that donor disease state affects cell morphology.

### 3.2. T cells from both healthy donors and CLL patients respond to substrate stiffness

Turning to the extrinsic factor of extracellular stiffness, T cells from both healthy and CLL donors exhibited changes in Area and Roundness as a function of substrate modulus (Figure 2B). However, the mechanosensing effect was more pronounced for cells from healthy donors than CLL patients, suggesting a functional impact of disease state and exhaustion on this response.

While these experiments demonstrate an impact of disease state and substrate stiffness on two morphological features (Area and Roundness), what we seek is a way to identify the experimental parameters associated with a sample from cell morphology. The differing responses of Area and Roundness on disease state and substrate stiffness make the use of either feature individually complicated for this purpose. As such, the next sections expand this approach to include additional morphological features.

### 3.3. PCA reveals the variance between CLL and Healthy T cells and identifies important morphological features contributing to the variance

**Figure 3.**
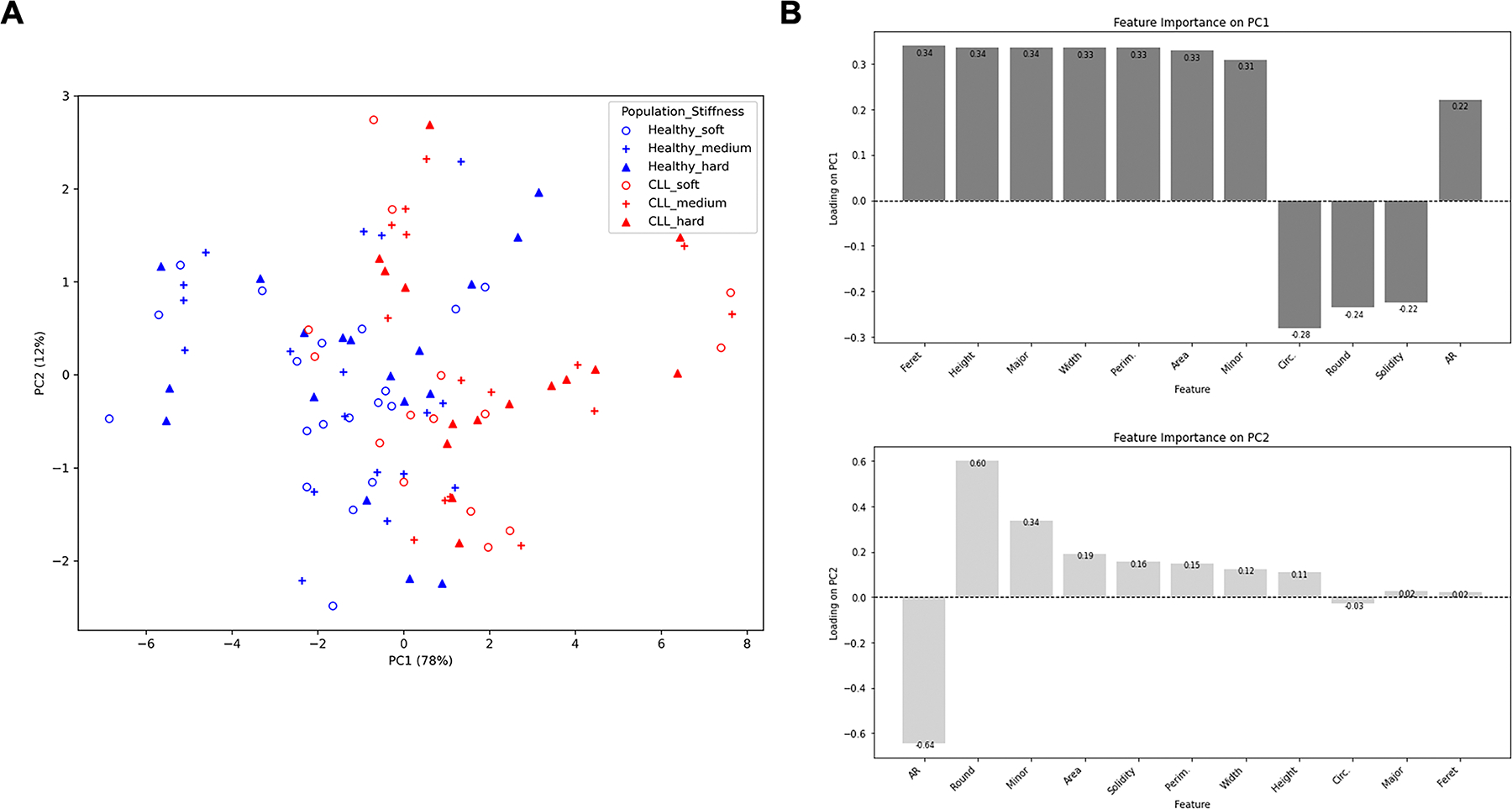
PCA reveals the variance between CLL and Healthy T cells and identifies important morphological features contributing to the variance. (A) Two-dimensional representation of PCA analysis. Projection of the data along PC1 showed a separation between Healthy (blue) and CLL (red). Three stiffness conditions which the data were derived from were also shape-coded. Each data point represents an individual sample. (B) Feature importance on PC1 and PC2.

A dataset containing 11 morphological features was generated from T cells as a function of disease state and substrate stiffness. Dimensionality reduction of this data by Principal Component Analysis (PCA) revealed a first component (PC1) that strongly expressed the dataset, accounting for 78% of the variance; PC2 was associated with 12%, leaving 10% to the remaining principal components (Figure 3A). Examination of PC1 identified a set of key features with similar and large weights, including Area, Feret’s Diameter, and Minor (Figure 3B). These weights also captured the anti-correlation between Area and Roundness suggested in Figure 2. However, the lower weight of Roundness in PC1 does suggest that these two parameters are not simply anti-correlated, and they may provide additional information useful in identifying the intrinsic and extrinsic conditions. Notably, the projection of the data along PC1 showed the separation of datasets based on disease state (Figure 3A, red vs. blue).

### 3.4. Machine learning classifies CLL patients based on morphological features

While PCA analysis showed the promise of using multiple features to distinguish between intrinsic and extrinsic parameters, there are several limitations to this approach. Most prominently, the identification of cell spreading conditions from morphological data is a classification rather than an analysis question. In addition, PCA is fundamentally a linear analysis approach, while the relationship between morphological inputs and the experimental conditions is likely more complex. Consequently, this section applies machine learning tools to the classification of cells, focusing first on the disease state. Three different classification models were evaluated and compared through the average performance of three independent runs of 10-fold cross-validation (Table 1).

The first model used a Decision Tree with a single feature (Area) as input, resulting in an accuracy of 0.677, an Area Under the Curve (AUC) of 0.683, and a Mathew’s correlation coefficient (MCC) of 0.372. The second model utilized a Decision Tree with 11 morphological features as input, achieving an accuracy of 0.651, an AUC of 0.650, and a significantly improved MCC of 0.649. The third model employed a Random Forest with all 11 morphological features as input, demonstrating an improved accuracy of 0.753, a notably higher AUC of 0.812, and an MCC of 0.596. This comparison indicates that incorporating multiple morphological features plays an important role in classifying T-cell disease states.

Furthermore, we investigated the impact of including substrate stiffness as an additional input along with the 11 morphological features to improve the classification accuracy. Two methods of labeling stiffness were employed, including one-hot encoding and normalized Young’s modulus (Table 2, 3). Incorporating stiffness as an additional input led to an improvement across all models, with one hot encoding providing similar performance to inclusion of modulus as a numeric variable.

### 3.5. Effect of cytoskeletal protein inhibitors on T cell response to substrate stiffness

**Figure 4.**
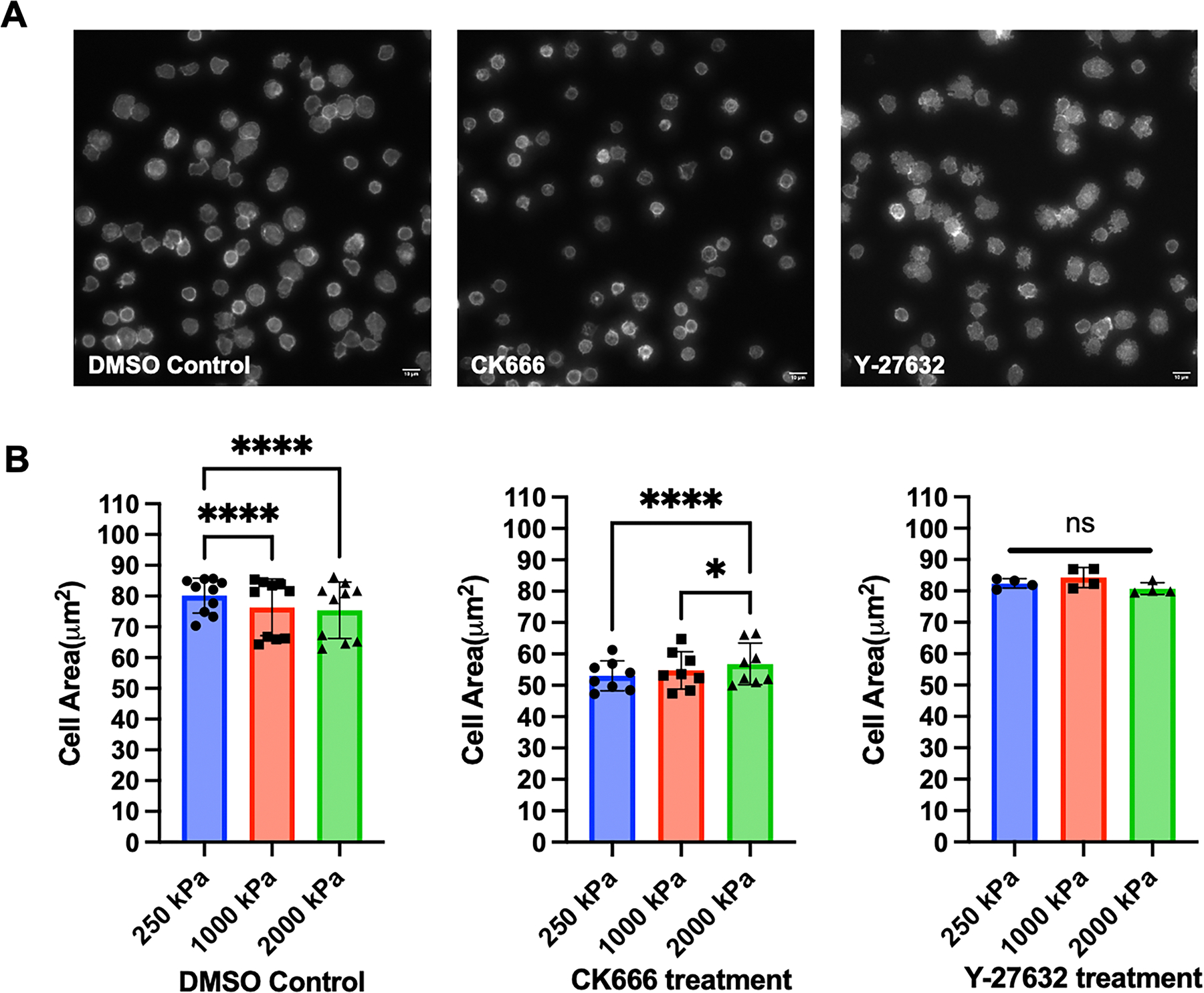
Effect of cytoskeletal protein inhibitors on T cell mechanosensing. (A) T cells from a healthy donor were treated with DMSO control, CK666 (100 μM), or Y-27632 (60 μM) for 15 min before being seeded onto PDMS substrates, followed by fixation, permeabilization, and actin staining. Image examples (250 kPa substrate) were shown; scale bar: 10 μm. (B) Quantitative analysis reveals the effect of CK666 and Y-27632. Data are mean ± s.d. For DMSO, n=10; for CK666, n=8; for Y-27632, n=4. Statistical significance was determined using two-way ANOVA with Tukey multiple comparison test, *p<0.05, ****p<0.001.

Finally, we investigated the effects of cytoskeletal protein inhibitors to understand the contributions of different dynamics on cell morphology. Inhibition of Arp2/3-based actin branching using CK666 (100 μM) significantly reduced cell spreading. Inhibition of Rho-modulated actomyosin contraction using Y-27632 (60 μM) eliminated the mechanosensing response (Figure 4). These results show that different types of cytoskeletal dynamics, and their associated signaling pathways, are represented by the complex measurement of cell morphology.

## 4. Discussion

An individual’s immune response is a complicated result of multiple factors, including genetics, environment, disease, and lifestyle. This variability impacts cellular therapies based on immune cells, from the success of ex-vivo cell production to the specification of systems for activating immunity in situ. The ability to assess T cell functional response would be transformative to these therapies. The driving concept behind this study is that measures of complex cellular functions such as morphology provide insight into the state of immune cells to a degree not attainable through biomarkers and -omics based technologies; we previously showed that measures of cell migration provide a better predictor of subsequent function than biomarkers or clinical diagnoses.

This paper strives for a more rapid and deployable approach to describing T cell function, focusing on cell morphology. Most directly, we show that donor disease state and cell response to substrate stiffness influence cell morphology. Conversely, we show that machine learning approaches combining multiple quantitative measures of morphology have promise in identifying the impact of disease state on an individual’s T cells. Applied to the clinical setting, this approach promises a measure of how exhausted or CLL-like an individual’s cells are following therapy, which could guide subsequent treatment. Data from additional donors with known disease outcomes in response to treatment will be needed for such an assay, and will be the subject of further studies.

Machine learning-based morphology analysis was less effective in identifying what stiffness of material was used to stimulate the cells. Continued refinement of this model could allow specification of biomaterial properties that optimize *ex vivo* or *in situ* activation of T cells, avoiding time- and resource-consuming trial-and-error approaches [31]. Notably, the analysis workflow used all 11 measures of cell morphology that were collected. Many of these features – such as Major, Minor, Aspect Ratio, and Feret’s Diameter – seem to capture similar aspects of cell spreading. It is tempting to remove measures that have some correlation from the analysis to improve accuracy. However, each of these measures has a specific definition that is distinct from the others. While not fully independent, the inclusion of all parameters to the machine learning workflow has the best opportunity to optimize performance, given sufficient data. Conversely, the inclusion of other measures of morphology that capture features very different from the existing list may improve performance. Finally, we anticipate that further development of these methods, such as using image-based deep-learning tools, may improve the performance of this approach [32].

## Supplementary Material

Supplementary material

Figure S1

Figure S2

## Figures and Tables

**Table 1.** Only morphological features as input.

	Single-Feature (Area) Decision Tree	Multi-Feature Decision Tree	Random Forest

Accuracy	0.677	0.651	0.753
AUC	0.683	0.650	0.812
Sensitivity	0.673	0.680	0.750
Specificity	0.693	0.616	0.753
MCC	0.372	0.649	0.596

**Table 2.** Stiffness as an additional input feature (one hot encoding)

	Single-Feature (Area) Decision Tree	Multi-Feature Decision Tree	Random Forest

Accuracy	0.729	0.693	0.765
AUC	0.735	0.694	0.846
Sensitivity	0.746	0.708	0.725
Specificity	0.725	0.680	0.819
MCC	0.409	0.549	0.580

**Table 3.** Stiffness as an additional input feature (normalized Young’s Modulus)

	Single-Feature (Area) Decision Tree	Multi-Feature Decision Tree	Random Forest

Accuracy	0.749	0.690	0.756
AUC	0.760	0.698	0.836
Sensitivity	0.749	0.670	0.713
Specificity	0.770	0.725	0.812
MCC	0.490	0.510	0.627
