# Supplementary material for: Assaying and classifying T cell function by cell morphology
Source: BioMedInformatics. Author manuscript; Available in PMC 2025 Jun 1. (PMC11542667; doi:10.3390/biomedinformatics4020063)
Supplement: Supplementary material [file NIHMS2027448-supplement-Supplementary_material.docx]

Supplementary Material


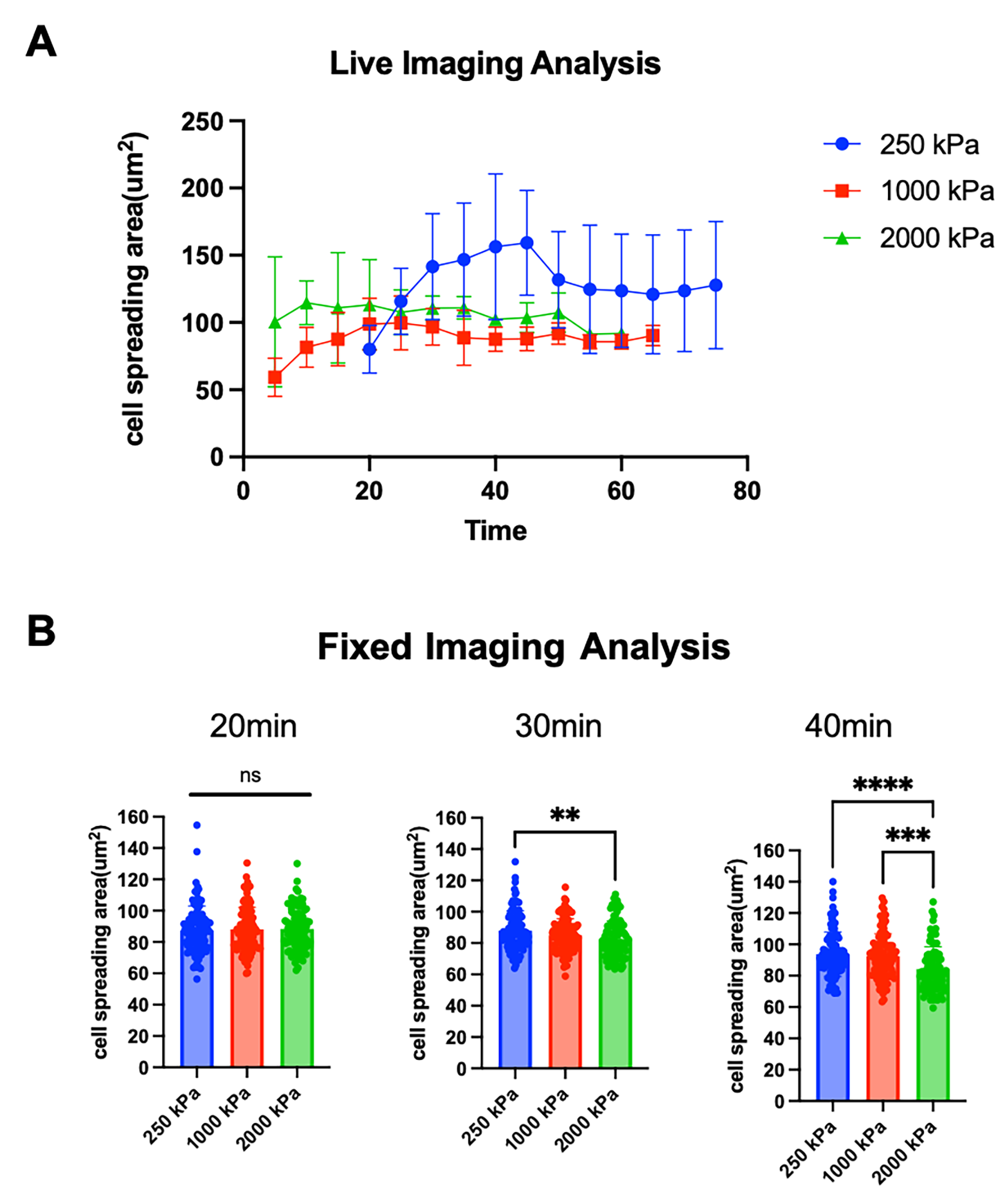


**Figure S1.** Dynamics of T cell spreading on PDMS substrates. (A) Live imaging captures T cell spreading over 60 min and identifies 40 min as a time point during which cells stabilize on each surface and produce the greatest difference as a function of elastic modulus. (B) Fixed imaging analysis comparing 20 min, 30 min, and 40 min timepoints. The 40 min timepoint retains the stability and resolution seen in the live-cell assays. Each data point stands for each cell. Statistical significance was determined using one-way ANOVA with Tukey multiple comparison test, **p<0.01, ***p<0.05, ****p<0.001.


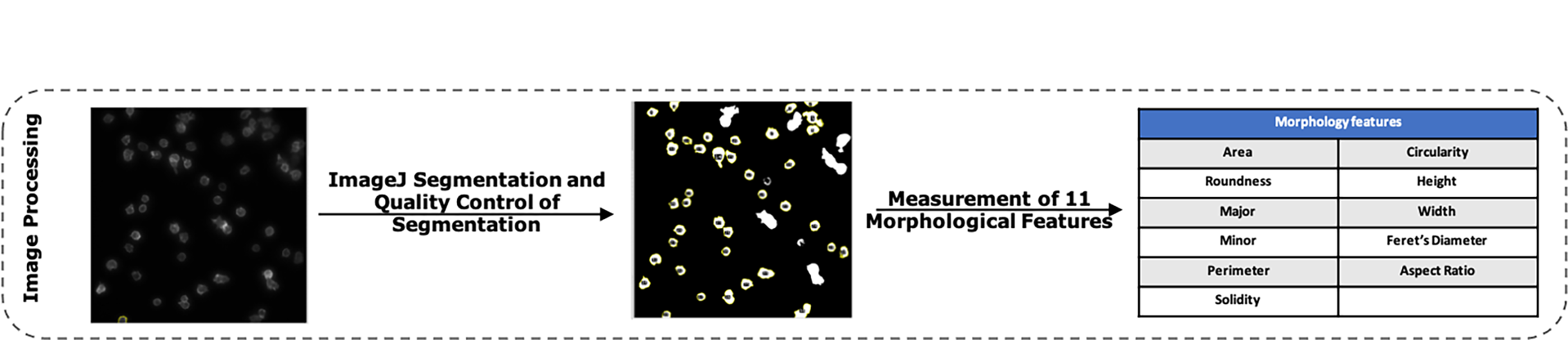


**Figure S2.** Imaging processing workflow. Fixed imaging was performed under 40X magnification to acquire more cells in a field of view for analysis. Image analysis was performed in ImageJ using functions of Smoothing, Thresholding, Set Measurement, and Analyze Particles to measure morphological features of single cells, including Area, Perimeter, Width, Height, Major, Minor, Circularity, Feret’s Diameter, Aspect Ratio (AR), Roundness, and Solidity. Only accurately segmented single cells were selected by quality control for further analysis.
