# Supplementary figures and images for: Assaying and classifying T cell function by cell morphology
Source: BioMedInformatics. Author manuscript; Available in PMC 2025 Jun 1. (PMC11542667; doi:10.3390/biomedinformatics4020063)

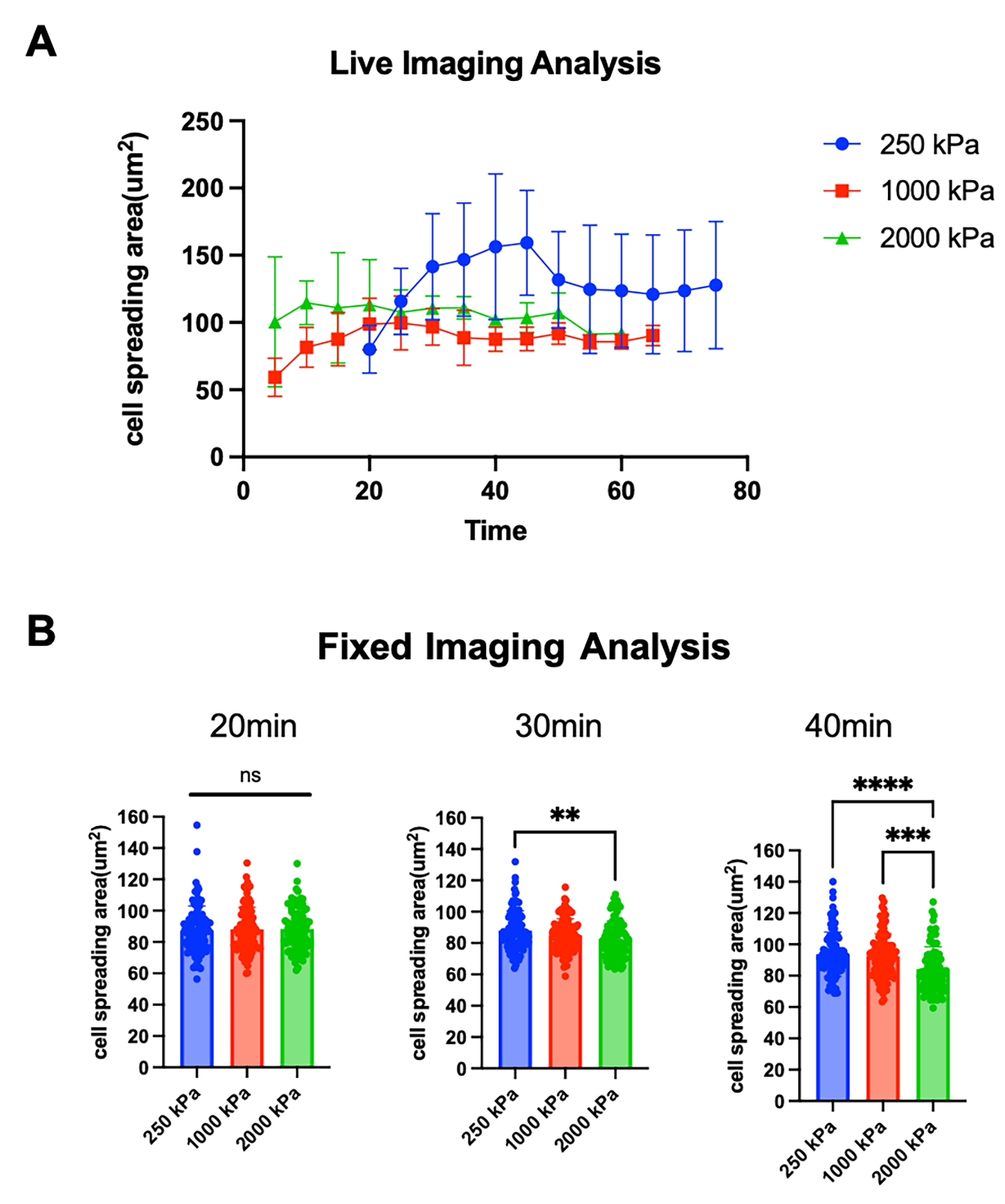

Supplement: Figure S1 [file NIHMS2027448-supplement-Figure_S1.png]

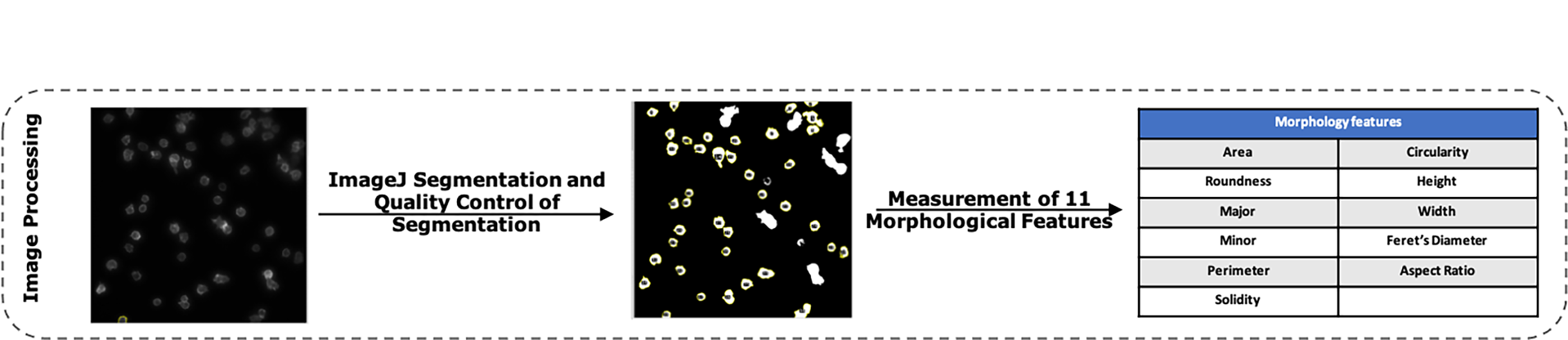

Supplement: Figure S2 [file NIHMS2027448-supplement-Figure_S2.png]
